# Evaluation of the Reliability and Quality of YouTube Videos on Ovarian Cysts

**DOI:** 10.7759/cureus.22739

**Published:** 2022-03-01

**Authors:** Cengiz Andan, Mustafa F Aydin

**Affiliations:** 1 Obstetrics and Gynecology, TC Ministry of Health, Health Sciences University Gazi Yasargil Diyarbakır Training and Research Hospital, Diyarbakir, TUR

**Keywords:** gqs, discern, internet, youtube, ovarian cyst

## Abstract

Aim

The aim of this study is to investigate the quality and reliability of YouTube videos containing content related to ovarian cysts.

Methods

The search terms “Ovarian Cyst”, “Ovarian Cyst Symptoms”, “Ovarian Cyst Treatment”, and “Ovarian Cyst Diagnosis” were searched on YouTube. A total of 110 videos were examined and repetitive videos, non-English videos, videos with advertising content, videos with entertainment and news content, and videos with very poor image and sound quality were excluded. Finally, the videos included in the study were evaluated using DISCERN and Global Quality Scale (GQS).

Results

It was found that 50 videos examined in this study were uploaded between the years 2014 and 2020, with an average of 492.252 ± 710.768 and a total of 24.612.595 views. The mean DISCERN score given to 50 videos analyzed by two researchers was 2.81 ± 1.3 and the mean GQS score was 2.88 ± 1.4. When we divided the scores given to the videos by two researchers into three groups, it was determined that 27 (54%) of the 50 videos were in the misleading/poor quality group, nine (18%) were in the medium quality group, and 14 (28%) were in the useful/quality group.

Conclusion

It has been determined that the videos with "ovarian cyst" content on YouTube are generally of poor quality. Bad quality videos were uploaded by non-doctors and attracted more attention than videos uploaded by doctors.

## Introduction

An ovarian cyst is a sac filled with fluid or semi-fluid material that arises in one of the ovaries. The number of diagnosed ovarian cysts has increased due to regular examinations and advances in ultrasound technology [1]. Although most patients with ovarian cysts are asymptomatic, some cysts are associated with a range of symptoms, and those that are malignant often do not cause symptoms until the advanced stage [2].

Although ovarian cysts are frequently seen in women between the ages of 20 and 45, they can occur at any age from the neonatal period to the post-menopausal period. These cysts are usually detected incidentally during an ultrasound or pelvic examination. These cysts are usually small in size and self-limiting [3]. However, it is known that the detection of ovarian cysts causes anxiety in women [4]. These patients often turn to other resources for finding solutions and sharing experiences and worries before seeking medical help.

In studies, it is reported that patients and their relatives primarily search for information about their health on the Internet and do not share this information with their doctors [5-6]. At the top of these websites are platforms such as Google (www.google.com) and YouTube (www.youtube.com). These platforms contain very educational and useful information, as well as highly misleading and harmful information. However, the usefulness of this information cannot be distinguished by the patients and their relatives. YouTube is the world's largest video-sharing website and is visited by millions of users daily. According to YouTube statistics, there are over 2 billion YouTubers globally, which corresponds to approximately one-third of all Internet users on a global scale. In addition, considering that approximately one billion hours of views are made per day, it is clear how widely used YouTube is around the world [7]. In many studies evaluating the quality of health-related YouTube videos, it has been reported that the quality of the videos examined, especially those uploaded by non-doctor users, is generally poor or moderate [8-14]. However, in our literature review, we did not find a study in which ovarian cyst-containing videos on YouTube were examined. The aim of this study is to investigate the quality and reliability of ovarian cyst content videos on YouTube.

## Materials and methods

Ethical disclosure

Since only public-access data was used in this study, it was exempted from ethics committee approval.

Study design

This study was designed by two gynecologists who are experts in their field on 01/06/2021. The search terms to be used for the research were determined using the Google Trends (https://trends.google.com) application. A search was made by entering the term "Ovarian Cyst" in the Google Trends application and selecting "Entire World" and the last five years from the filters. In our study, the search terms “Ovarian Cyst”, “Ovarian Cyst Symptoms”, “Ovarian Cyst Treatment”, and “Ovarian Cyst Diagnosis” were used with both the results of the Google Trends application and the consensus of the two researchers. In addition, a Microsoft Excel file (Microsoft Corporation, Redmond, WA) was prepared by the researchers in order to save the data. In this Excel file, the video link, uploader's quality, video's content, length (minutes), total number of views, date it was uploaded, date it was viewed, time since the video was uploaded, number of comments, likes, and dislikes, and Video Power Indexes (VPI) have been recorded. Finally, the study design was created by deleting the past searches and cookies of the computer on which the research was to be conducted.

Data collection

Two researchers searched for the determined search terms and used the "relevance" and "view counts" filters. For each search term, the most relevant and most viewed videos on the subject were examined and the information of all the videos included in the study was saved in an Excel file. In the study, a total of 110 videos were examined and repetitive videos, non-English videos, videos with advertising content, videos with entertainment and news content, videos with very poor image and sound quality were excluded. The 50 most relevant and most viewed videos that fit the study sample were included in the study.

Evaluation of the data

The 50 videos included in the study were evaluated by the researchers in separate environments using the Quality Criteria for Consumer Health Information (DISCERN) [15] and Global Quality Scale (GQS) [16], which were previously used in many YouTube studies [8-9,12,14]. The structured DISCERN scale is used to determine the reliability of videos. The scale consists of five questions in total, showing that 1-2 points are bad, 3 points are medium, 4 points are good, and 5 points are perfectly reliable. Similarly, the GQS scale consists of five questions and shows the quality of the videos. The questions of the DISCERN and GQS scale are given in Table 1.

**Table 1:** Questions related to DISCERN and GQS GQS: Global Quality Scale

SCORES	DISCERN
1	Is the video clear, concise, and understandable?
2	Are reliable sources of information used?
3	Is the information presented balanced and unbiased?
4	Are additional sources of information listed for patient reference?
5	Are areas of uncertainty/controversy mentioned?
SCORES	GQS
1	The video is of poor quality, poor flow, lacks most information, and is, therefore, not useful for patients.
2	The video is generally of poor quality, and although some information is given, it is of limited use for patients.
3	The video is of moderate quality, and some important information is sufficiently discussed. In these videos, accurate and incorrect information is presented in a balanced manner. However, high-quality information is provided together with misleading information.
4	The video is of good quality and good flow. The video is useful for patients, covering the most relevant information and presenting accurate information to a large extent, but it may include minor deficiencies.
5	The video is of excellent quality and excellent flow and is very useful for patients. These videos include completely accurate information

According to the scores given to the videos by the two researchers, videos with one to two points were classified as misleading/harmful videos, videos with three points as medium quality/safe videos, and videos with four to five points as useful/quality videos.

Statistical analysis

The data obtained in the study were statistically analyzed using the Statistical Package for the Social Sciences version 20.0 software (IBM Corp., Armonk, NY). Descriptive data are expressed as numbers, percentages, and median (minimum-maximum) values. The conformity of the data to the normal distribution was analyzed with the Shapiro-Wilk test. The agreement between the two researcher physicians was evaluated using the Kappa coefficient. A p-value of < 0.05 was considered statistically significant.

## Results

It was found that 50 videos examined in this study were uploaded between the years 2014 and 2020, with an average of 492.252 ± 710.768 and a total of 24.612.595 views. It was determined that the average number of views per day was 506.82 ± 601. It was also determined that eight (16%) of the videos were animated and 42 of them were real images. The data on the general characteristics of the videos are given in Table 2.

**Table 2:** Data on the general characteristics of videos

	n (%)	n (%)	View	Comment	Like	Dislike
Image						
Real	8 (16)	19.649.132	1.725	39.589	1.208	92.60 ± 3.3
Animation	42 (74)	16.274.401	20.392	198.184	8.396	94.10 ± 4.8
Uploaders						
Physician	15 (30)	15.443.085	10.148	18.440	6773	94.34 ± 5.4
Hospital Channel	4 (8)	985.324	558	4.071	250	94.46 ± 1.2
Health Channel	5 (10)	2.412.236	3.757	13.609	1.076	94.17 ± 3.7
Patients	13 (26)	401.057	3.005	5.009	263	93.27 ± 5.4
Herbalist	2 (4)	2.546.883	6.181	43.500	2.470	88.12 ± 7.3
Blog Channel	5 (10)	9.402.046	4.875	99.100	4.973	94.53 ± 3.3
Yoga Channel	6 (12)	4.718.912	5.667	73.000	2.261	94.84 ± 4.6
Content						
General Info	10 (20)	2.752.411	2.088	12.653	871	93.34 ± 3.2
Surgical Technique	7 (14)	1.712.328	931	8.021	681	93.38 ± 4.2
Patient Experience	14 (28)	407.312	3.008	5.022	263	93.77 ± 5.4
Yoga	5 (10)	3.926.829	4.164	69.600	1.697	95.54 ± 4.6
Herbal	9 (18)	10.371.599	15.079	147.800	7.311	93.5 ± 4.6
Symptom	2 (4)	14.791.616	7.467	259.000	6.100	96.76 ± 1.2
Diet	3 (6)	1.948.448	1.456	12.800	1.143	93.54 ± 2.7

It was determined that the average length of the 50 videos examined was 8.25 ± 6.1 minutes, the average daily viewing rate was 506.82 ± 855.38, and the average time elapsed since the videos were uploaded was 1.172 ± 583 days.

**Figure 1:**
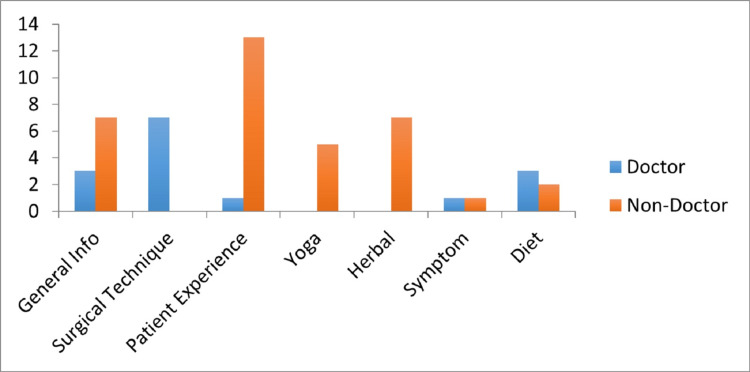
Distribution of uploaded videos by content

When we divided the quality of the video uploaders into two groups - doctors and non-doctors, it was found that 15 videos were uploaded by doctors and 35 videos were uploaded by non-doctor users. The distribution of the videos uploaded by doctors and non-doctor users according to the content is given in Figure 1.

The mean DISCERN score given to 50 videos analyzed by two researchers was 2.81 ± 1.3 and the mean GQS score was 2.88 ± 1.4. The mean DISCERN score of the first investigator was 2.86 ± 1.2 and the mean GQS score was 2.94 ± 1.4. The mean DISCERN score of the second investigator was 2.76 ± 1.3, and the mean GQS score was 2.82 ± 1.3. The average DISCERN score given by the researchers to videos uploaded by doctors was 4.43 ± 1.27, and the mean GQS score was 4.65 ± 1.42. The mean DISCERN score of non-doctor uploaders was 2.11 ± 1.09, and the mean GQS score was 2.12 ± 1.26. The scores given by the researchers to the videos according to both DISCERN and GQS are given in Table 3.

**Table 3:** Distribution of researchers' scores on DISCERN and GQS GQS: Global Quality Scale

	n (%)	DISCERN 1	DISCERN 2	GQS 1	GQS 2
Image		Mean ± SD			
Real	8 (16)	2.75 ± 1.3	2.62 ± 1.4	3.12 ± 1.2	3 ± 1.3
Animation	42 (74)	2.88 ± 1.2	2.78 ± 1.3	2.90 ± 1.4	2.76 ± 1.3
Uploaders					
Physician	15 (30)	4.4 ± 1.2	4.46 ± 1.3	4.71 ± 1.4	4.53 ± 1.4
Hospital Channel	4 (8)	2.75 ± 1	3.0 ± 1	3.25 ± 1	3 ± 1
Health Channel	5 (10)	3.4 ± 1.4	3 ± 1.1	3.2 ± 1.1	3.2 ± 1.3
Patients	13 (26)	2 ± 1.5	1.84 ± 1.3	1.46 ± 1.4	1.53 ± 1.3
Herbalist	2 (4)	2 ± 1.3	1.5 ± 1.2	2.5 ± 1.2	2 ± 0.8
Blog Channel	5 (10)	1.6 ± 1.2	1.4 ± 1.5	2 ± 1.7	2 ± 1
Yoga Channel	6 (12)	1.83 ± 1.2	1.66 ± 1.3	2.16 ± 1.1	1.83 ± 1.13
Content					
General Info	10 (20)	3.5 ± 0.8	3.5 ± 0.7	3.8 ± 0.8	3.7 ± 0.9
Surgical Technique	7 (14)	4.28 ± 1.2	4.22 ± 1.3	4.85 ± 1.4	4.42 ± 1.3
Patient Experience	14 (28)	2.21 ± 1.1	2 ± 1.3	1.71 ± 1.4	1.71 ± 1.3
Yoga	5 (10)	2 ± 1.2	1.6 ± 1.3	2.2 ±1.1	1.8 ± 1.4
Herbal	9 (18)	2 ± 1.8	1.57 ± 1.3	2.28 ± 1.1	2 ± 1
Symptom	2 (4)	2.5 ± 1.5	3 ± 1	3 ± 1	3 ± 1
Diet	3 (6)	2 ± 1.2	1.6 ± 1.3	2.2 ± 1.1	1.8 ± 1.4

When we divided the scores given to the videos by two researchers into three groups, it was determined that 27 (54%) of the 50 videos were in the misleading/poor quality group, nine (18%) were in the medium quality group and 14 (28%) were in the useful/quality group. The quality data of the videos according to the image type, the quality of the uploaders, and the content are shown in Table 4.

**Table 4:** Distribution of videos by quality groups

	n (%)	Misleading/Harmful	Medium quality/Safe videos	Useful/Quality
Image				
Real	8 (16)	4	2	2
Animation	42 (74)	23	7	12
Uploaders				
Doctor	15 (30)	0	5	10
Hospital Channel	4 (8)	1	1	2
Health Channel	5 (10)	0	3	2
Patients	13 (26)	13	0	0
Herbalist	2 (4)	2	0	0
Blog Channel	5 (10)	5	0	0
Yoga Channel	6 (12)	6	0	0
Content				
General Info	10 (20)	0	5	5
Surgical Technique	7 (14)	0	2	5
Patient Experience	14 (28)	13	0	1
Yoga	5 (10)	5	0	0
Herbal	9 (18)	9	0	0
Symptom	2 (4)	0	0	2
Diet	3 (6)	0	2	1

When Table 4 is examined, it is seen that all of the poor quality/misleading videos were uploaded by non-doctor users. It was determined that five of the 15 videos uploaded by the doctors were of medium quality and 10 of them contained useful and quality information. However, videos uploaded by non-doctors were found to be viewed and liked more than videos uploaded by doctors, even though they contain misleading information.

When the DISCERN and GQS scores of the two researchers were analyzed by correlation analysis, a statistically significant and strong correlation was found between researchers in terms of both DISCERN and GQS scores. However, a perfect agreement was found between the two researchers (Table 5).

**Table 5:** Correlation analysis among researchers GQS: Global Quality Scale

	Mean ± SD	p	r	Cronbach α
DISCERN 1	2.86 ± 1.2	p<0.01	0.928	0.946
DISCERN 2	2.76 ± 1.3			
GQS 1	2.94 ± 1.4	p<0.01	0.891	0.912
GQS 2	2.82 ± 1.3			

## Discussion

YouTube is the world's most-visited video-sharing platform. There are educational, entertaining, and useful videos on this platform. However, the quality of videos, especially with health content, is worried by experts. For this reason, health-related videos on YouTube have been reviewed by experts, and it has been reported that these videos are generally of bad or medium quality [8-10,13-14,17-19]. In this study, in which the quality and reliability of the videos with ovarian cyst content on YouTube were examined, it was found that 27 of the videos were of bad quality and poor quality, nine of them were of medium quality, and 14 of them were of good quality and reliability.

It was determined that the 50 videos examined were viewed 24,612,595 times in total. In a study examining 66 videos containing hysterectomy, it was reported that the total number of views was 4.679.118 [18]. In a study evaluating 52 videos with contraceptive implant content on YouTube, it was reported that the total number of views was 2.221.118 [19]. The differences between the number of views may vary according to the research subjects. In our study, it was determined that the videos uploaded by doctors were viewed 4.145.137 times and the videos uploaded by non-doctors were viewed 20.467.458 times. It was found that the videos uploaded by doctors were liked 44.181 times and the videos uploaded by non-doctors were liked 238.239 times. In many previous studies, it has been reported that videos uploaded by patients and other users are viewed and liked more [9-10,13,18].

It was determined that 54% (n=27) of the 50 videos examined in our study were of poor quality and had misleading content. Of these misleading and poor-quality videos, 13 (26%) were uploaded by patients and their relatives, six (12%) by yoga channels, five (10%) by blog channels, and two (4%) by herbalists. Similar results have been previously reported in studies that examined YouTube videos [18-19]. In the study in which hysterectomy videos were analyzed, it was reported that 51% of the videos contained misleading information and were of poor quality [18]. In another study evaluating gynecological physical examination videos, it was reported that 65% of the videos examined contained bad quality and misleading information [20].

When we examine the poor quality videos according to their content, it has been determined that all of the videos with patient experience, herbal treatment, and yoga have bad quality/misleading content. In the videos shared by the patients and their relatives, patient experiences were conveyed. These videos contain incomplete and incorrect information and were evaluated as bad/misleading by two studies. In many previous studies, it has been reported that the videos uploaded by patients have misleading/harmful content [9-14,20-21]. We think that these videos uploaded by patients should be organized with the cooperation of healthcare professionals and patients, and these videos should be shot in the attendance of experts. In addition, it was determined in our study that yoga, exercise, and herbal treatment methods also contain misleading/harmful information. These videos have been viewed more than 13 million times and have been liked 194,609 times. In similar studies, it is reported that videos with such content are viewed and liked more [8,13-14,19,21].

It was determined that 18% (n=9) of the videos examined in our study were medium quality and 28% (n=14) were good quality/useful videos. Of the medium-quality videos, five were uploaded by doctors, three by health channels, and one by the hospital channel. While 10 of the good-quality videos were uploaded by doctors, two videos were uploaded by hospital channels, and two videos were uploaded by health channels. In previous similar studies, it was reported that useful videos were uploaded by doctors and health institutions [18-22]. However, in many studies, videos uploaded by doctors or healthcare organizations have also been reported to be of poor or moderate quality [10-11,13,17]. Researchers reported that health videos uploaded by experts on major platforms such as YouTube must undergo an absolute peer review [8-14].

In our study, when we divided the video uploaders into two groups - doctors and non-doctors, both the DISCERN and GQS scores of the videos uploaded by the doctors were statistically significantly higher than the non-doctors (for all, p<0.05). In addition, a perfect agreement was found between the scores given by both DISCERN and GQS between the two researchers.

There have been some studies evaluating gynecology and obstetrics videos on YouTube. In a study evaluating 66 videos containing hysterectomy, it was reported that only 6% of the videos were of excellent quality [18]. In the study by Erdogan, 50 videos were examined and 18% of the videos were reported to be of excellent quality [11]. In another study examining 176 videos of gynecology examinations on YouTube, it was reported that 34.5% of the videos were of excellent quality [20]. In our study, 28% of the videos reviewed were found to be of excellent quality. These results show that YouTube is not a safe source for obstetrics and gynecology videos.

Limitations of the study

The primary limitation of the study is that only English videos were analyzed. It should also be noted that the video parameters on YouTube may change. The fact that we examined 50 videos in our study can be considered a limitation. However, for these 50 videos, four search terms were used and selected from 110 videos using the "relevance" and "most viewed" filtering methods. To the best of our knowledge, our study is the first to examine videos containing ovarian cysts.

## Conclusions

As a result, it has been determined that videos with "ovarian cyst" content on YouTube are generally of poor quality. Bad quality videos were uploaded by non-doctors and attracted more attention than videos uploaded by doctors. We think that health-related videos should be shared by health professionals and that these videos should be peer-reviewed. In addition, in these videos shared by experts, clear information should be given instead of academic language, in a way that the viewers can understand. Finally, we believe that health professionals should share not only about diseases but also about the harms of such misleading videos.
